# Impact of resistance training and chicken intake on vascular and muscle health in elderly women

**DOI:** 10.1002/jcsm.13572

**Published:** 2024-11-21

**Authors:** Shumpei Fujie, Naoki Horii, Hiroki Kajimoto, Henry Yamazaki, Kenichiro Inoue, Keiko Iemitsu, Masataka Uchida, Takuma Arimitsu, Yasushi Shinohara, Kiyoshi Sanada, Motohiko Miyachi, Motoyuki Iemitsu

**Affiliations:** ^1^ Faculty of Sport and Health Science Ritsumeikan University Shiga Japan; ^2^ Research Fellow of Japan Society for the Promotion of Science Tokyo Japan; ^3^ Faculty of Health Care, Undergraduate Department of Human Health Hachinohe Gakuin University Aomori Japan; ^4^ Faculty of Sport Sciences Waseda University Saitama Japan

**Keywords:** Angiotensin II, Arterial stiffness, Elderly, High‐protein, Resistance training

## Abstract

**Background:**

Resistance training is a well‐known exercise therapy for preventing and improving lacks of muscle mass, strength, and quality with advances in age; however, its effects on arterial stiffness are not beneficial. Additionally, a higher intake of protein, which is an effective nutrient for muscle health, results in lower arterial stiffness. Whether the combination of moderate to high‐intensity resistance training and high‐protein intake would improve muscle mass, strength, and quality and cancel the resistance training‐induced increase in arterial stiffness in elderly women remains unclear.

**Methods:**

Ninety‐three elderly women (67.2 ± 5.3 years) were randomly divided into four groups; sedentary control (CON), higher dietary animal protein intake (HP), resistance training (RT), and combination of RT and HP (RT + HP) groups. Participants in the RT and RT + HP groups completed 12 weeks of resistance training (exercise intensity at 70% of one‐repetition maximum (1‐RM), three sets with 10 repetitions of leg extension and curls, 3 days/week). In addition to the daily diet, the HP and RT + HP groups consumed steamed chicken breast as a high‐protein diet.

**Results:**

Percent changes in thickness (indices of muscle mass) and echo intensity (index of muscle quality) in the quadriceps muscle, 1‐RM of leg extension and curls (index of muscle strength), and circulating C1q levels (a potential biomarker of muscle fibrosis) in the RT and RT + HP groups significantly improved after both RT and RT + HP interventions (*P* < 0.05). Percent changes in carotid‐femoral pulse wave velocity (cfPWV) and carotid β‐stiffness (indices of arterial stiffness), and circulating angiotensin II (a vasoconstrictor peptide hormone) levels via each intervention were significantly higher in the RT group (4.9 ± 12.7%, 13.8 ± 13.5%, 94.9 ± 132.7%, respectively), as compared with the CON group (−2.5 ± 5.9%, 0.2 ± 8.1%, 21.2 ± 79.3%, respectively) (*P* < 0.05). Of note, no significant differences in the cfPWV, carotid β‐stiffness, and circulating angiotensin II levels between the RT + HP (−2.4 ± 9.3%, 2.4 ± 10.3%, −5.7 ± 29.6%, respectively) and CON groups were observed. Furthermore, significant positive relationships between the percent changes in circulating angiotensin II levels, and cfPWV (*r* = 0.438, *P* < 0.01) and carotid β‐stiffness (*r* = 0.328, *P* < 0.01) were observed.

**Conclusions:**

The combination of moderate to high‐intensity resistance training and regular intake of steamed chicken breast as a high‐protein food could increase muscle mass, strength, and quality and could cancel resistance training‐induced increases in arterial stiffness in elderly women.

## Introduction

A substantial loss of muscle mass, strength, and quality (sarcopenia) occurs with advancing age,^1^ along with the development and progression of cardiovascular disease (CVD), which is the primary cause of overall mortality worldwide.^2^ Reduced skeletal muscle mass, strength, and quality are important risk factors for CVD and are closely related to arterial stiffening.^3^ A meta‐analysis suggested that lower muscle mass was associated with higher pulse wave velocity (PWV) as an index of arterial stiffness, which is an independent risk factor for CVD.^4^ Thus, the deterioration of muscle health status is related to the vascular status.

Resistance training is a well‐known exercise therapy for the prevention and improvement of sarcopenia in elderly individuals. For instance, 12 weeks of high‐intensity resistance training (70–80% one‐repetition maximum [1‐RM]) increased the 1‐RM of a leg press and quadriceps cross‐sectional area (CSA) in elderly individuals.^5^ Additionally, in elderly women, high‐volume strength training improves echo intensity (EI), which is evaluated by measuring intramuscular adiposity and fibrosis,^6^ as an index of muscle quality, along with improvements in muscle mass and strength.^7^ However, the effects of resistance training on arterial stiffness are controversial. Regarding the effect of muscle training on arterial stiffness, several months of high‐intensity resistance training (80% 1‐RM) increase arterial stiffness and decrease arterial compliance in young adults.^8^ Furthermore, resistance training with a gradual increase in exercise intensity in each 4‐week period of a 12‐week program (low [30–50% 1‐RM], to medium [50–70% 1‐RM], to high [≥70% 1‐RM]‐intensity) increased arterial stiffness in older adults.^9^ Thus, despite the need to improve both muscle loss and CVD risk, high‐intensity resistance training has more beneficial effects on skeletal muscle but might not have vascular benefits. Therefore, new strategies are needed to address the aging‐induced loss of muscle mass, strength, and muscle quality, and the resulting in increased arterial stiffness.

It is well known that protein (especially the essential amino acids) is a key nutrient for muscle health in the elderly.^10^ In addition, the effects of daily intake of proteins and several amino acids on vascular health have recently drawn attention. Higher intake of several amino acids is associated with lower arterial stiffness in women, including young‐to‐older adults.^11^ Furthermore, a recent review concluded that there was increasing evidence that whey proteins, as well as bioactive peptides and amino acids released during digestion, can have beneficial effects on indices of arterial stiffness and endothelial function, and thus contribute to CVD risk reduction.^12^ Poultry meat, which is a well‐known good‐quality protein source with a low‐fat content, is consumed worldwide, especially in the United States and Europe.^13^ Among several high‐protein foods, the regular intake of poultry has beneficial effects on the risk among several high‐protein foods. Indeed, in 84,136 women, when compared with one serving of red meat per day, one serving of poultry per day was associated with a 19% decreased risk of coronary heart disease.^14^ Additionally, peptides from chicken breast are absorbed significantly faster than whey and soy proteins, making chicken breast a useful option for efficient protein intake to maintain and increase muscle mass.^15^ Thus, regular intake of chicken improves the health status of aged muscles and arteries, and consequently, may decrease CVD risk, for example, an index of arterial stiffness as an independent risk factor for CVD.

Herein, we hypothesized that the combination of resistance training and high‐protein intake would improve muscle mass, strength, and quality and cancel the resistance training‐induced increase in arterial stiffness in elderly women. To test our hypothesis, we assessed the effects of resistance training combined with regular intake of steamed chicken breast, a high‐protein food, on muscle mass, strength, quality, and arterial stiffness in elderly women.

## Materials and methods

### Study design and ethical approval

This was a parallel‐group (allocation ratio 1:1:1:1) randomized controlled trial (RCT). All participants were informed of the experimental procedures and risks, and provided written informed consent before participating in the study. Details of the clinical trial have been published previously,^16^ and this study is conducted as secondary analysis of the RCT. This study was approved by the Ethics Committee of Ritsumeikan University (BKC‐2018‐060) and conducted in accordance with the Declaration of Helsinki. The study was registered in the University Hospital Medical Information Network Clinical Trials Registry (UMIN‐CTR; UMIN000038253).

### Participants

**Figure 1:**
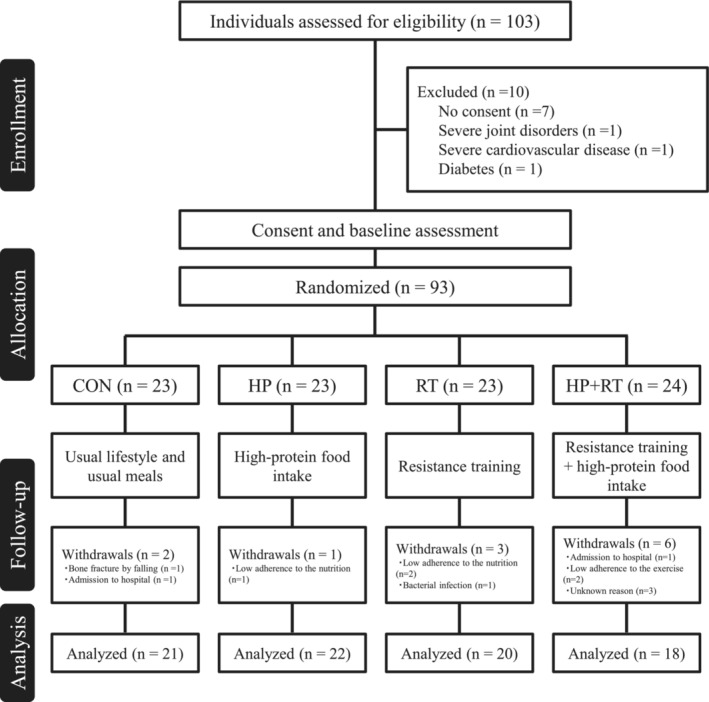
A flow chart showing the distribution of participants through the interventions.

All participants were recruited through newspaper advertisements in Otsu, Kusatsu, and Ritto City in Shiga, Japan. All participants were postmenopausal, and average postmenopausal years were 16.9 ± 9.2 years. About 50% of participants exercised habitually (above 30 min/day, 2 days/week for over 1 year), engaging in aerobic (walking, jogging, swimming, and so on), stretching, and resistance exercises, while <5% performed resistance exercises daily. The exclusion criteria were as follows: (1) <60 years of age; (2) secondary obesity due to adrenal gland disease, heart disease, gynecological disorder, joint disorder, mental disorder, severe liver dysfunction, cirrhosis, or severe renal dysfunction; and (3) patients who were going to the hospital for orthopedics or had limited exercise. Ten of the 103 participants were excluded because of the exclusion criteria and lack of consent, and 93 elderly women volunteered to participate. In this RCT, using computer‐generated random numbers, participants were randomly divided into four groups: sedentary control (CON; *n* = 23), high‐protein intake (HP; *n* = 23), resistance training (RT; *n* = 23), and a combination of RT + HP (RT + HP; *n* = 24). Since the 12 of 93 participants were withdrew because of several reasons during the follow‐up period, the data obtained from 81 participants (CON; *n* = 21, 67.6 ± 6.4 years, HP; *n* = 22, 67.0 ± 5.3 years, RT; *n* = 20, 66.9 ± 5.0 years, and RT + HP; *n* = 18, 67.2 ± 5.5 years) were analyzed as the final results (Figure 1). Each measurement and analysis were obtained by researchers who was blinded to the group assignment.

### Experimental design

Measurements in this study were performed before and after 12‐week of participation in either group, and included height, body weight, resting heart rate (HR), systolic blood pressure (SBP), diastolic blood pressure (DBP), carotid‐femoral PWV (cfPWV), carotid β‐stiffness, muscle thickness of the anterior (AT) and posterior (PT) thigh regions, quadriceps muscle cross sectional area (CSA), echo intensity (EI) in rectus femoris (RF), vastus lateralis (VL), and quadriceps femoris (QF) muscles, circulating levels of total cholesterol, high‐density lipoprotein (HDL) cholesterol, triglycerides, angiotensin II (Ang II), endothelin‐1 (ET‐1), complement component 1q (C1q), creatinine, and plasma renin activity (PRA). A more thorough description of each measurement can be found in Data S1. Fasting blood samples were drawn at least 48 h after the last resistance exercise session to avoid the influence of acute exercise effects. All participants were instructed not to eat or drink fluids, except water, for at least 10 h before reporting to the laboratory. Serum and plasma samples were centrifuged (1500× *g*, 15 min, 4°C) immediately after collection and stored at −80°C. All measurements were performed at a constant room temperature (24 ± 1°C).

### Resistance training intervention

Resistance exercise sessions were performed three times per week on alternate days for 12 weeks. Experienced trainers supervised all training sessions to ensure that proper techniques and progressions were used in each exercise session. Each session included two exercises: seated leg extensions and leg curls on weight‐stack machines (Life Fitness, Tokyo, Japan). The starting weight used during the resistance exercise portion of this study was set at 70% (moderate to high‐intensity) of each participant's predetermined 1‐RM for 3 sets of 10 repetitions using the machine. The rest period between the sets was 2 min. Before each resistance exercise session, participants walked on a treadmill for 3 min and stretched their lower limbs for 5 min as a warm‐up, they also performed a 5 min stretch as a cool‐down after each resistance exercise session. Measurements of 1‐RM were performed every 2 weeks, and the weight was increased for each participant to adjust the training weights to 70% of 1‐RM. Participants in the CON and HP groups were instructed not to change their activities of daily living during the 12‐week intervention period. All the participants were instructed to maintain their dietary habits throughout the study period. When the participation rate in 36 resistance training sessions in the 12‐week intervention was 90% or less, the individuals were excluded from the statistical analysis because of their ‘low adherence to the exercise intervention’.

### Intake of high‐protein food intervention

As a high‐protein food, a steamed chicken breast (Energy: 164 kcal; Protein: 22.5 g, Itoham Foods Inc., Hyogo, Japan) were used in this study. The participants in the HP and RT + HP groups consumed food three times/week on alternate days for 12 weeks. The participants in the CON and RT groups consumed carbohydrate‐rich food with energy similar to that of the high‐protein food three times/week on alternate days for 12 weeks. Additionally, the participants in the RT and RT + HP groups consumed the food within 30 min after each resistance exercise session, and the participants in the CON and HP groups consumed the food as a nosh between lunch and dinner. When participants consumed 90% or less of the 36 intakes of steamed chicken breast during the 12‐week intervention, the individual was excluded from the statistical analysis due to ‘low adherence to the nutrition intervention’.

### Statistical analysis

Data are expressed as means ± standard deviation (SD). Differences at baseline and percent changes in each parameter before and after each intervention among the four groups were assessed using one‐way analysis of variance (ANOVA). Bonferroni's post hoc test was applied in all cases where the measurements were significantly different. An unpaired t‐test was used for baseline comparisons of 1‐RM between the RT and RT + HP groups. Differences in percent changes in 1‐RM between groups and time points were assessed using two‐way repeated‐measures ANOVA. The relationships between each intervention‐induced percent change in any parameter were determined using Pearson's correlation coefficient. Statistical significance was set at *P* < 0.05. All statistical analyses were performed using StatView (version 5.0, SAS Institute, Tokyo, Japan), after confirming that all data were normally distributed using the Kolmogorov–Smirnov test. The minimum sample size for repeated‐measures ANOVA to examine comparisons among the four groups before and after each intervention with α set at 0.05 and power at 0.80 was 48 participants (12 in each group), as calculated by G*Power (ver. 3.1.9.6).

## Results

### Comparison of all parameters at baseline among the four groups

There were no significant differences in age, height, body weight, HR, SBP, DBP, cfPWV, β‐stiffness, muscle thickness in AT, muscle thickness in PT, quadriceps muscle CSA, EI in RF, EI in VL, EI in QF, circulating levels of total cholesterol, HDL cholesterol, triglycerides, Ang II, ET‐1, C1q, creatinine, and PRA at baseline among the four groups (Table 1). Furthermore, there were no significant differences in 1‐RM of leg extension or curl at baseline between the RT and RT + HP groups (Table 1).

**Table 1:** Comparison of characteristics at baseline among the four groups

	CON	HP	RT	RT + HP	*P* value
Age, years	67.6 ± 6.4	67.0 ± 5.3	66.9 ± 5.0	67.2 ± 5.5	0.9786
Height, cm	154.7 ± 6.9	155.7 ± 5.9	157.1 ± 4.1	153.6 ± 5.3	0.2739
Body weight, kg	54.3 ± 9.0	54.7 ± 8.7	57.0 ± 9.5	49.7 ± 7.5	0.0882
Muscle thickness in AT, cm	3.6 ± 0.5	3.8 ± 0.5	3.7 ± 0.6	3.6 ± 0.6	0.4891
Muscle thickness in PT, cm	5.0 ± 0.5	5.0 ± 0.8	5.0 ± 0.7	4.9 ± 0.8	0.9484
Quadriceps muscle CSA, %	55.7 ± 11.4	60.3 ± 9.4	59.7 ± 7.3	59.6 ± 4.8	0.4323
EI in RF, A.U.	140.4 ± 23.1	137.9 ± 23.0	148.2 ± 17.0	147.2 ± 18.4	0.3120
EI in VL, A.U.	127.2 ± 31.9	113.0 ± 31.4	113.6 ± 27.7	110.5 ± 32.6	0.3084
EI in QF, A.U.	133.8 ± 21.5	125.5 ± 25.1	130.9 ± 15.9	126.9 ± 25.3	0.6101
1‐RM of leg extension, kg	—	—	45.5 ± 11.2	44.9 ± 7.6	0.8526
1‐RM of leg curl, kg	—	—	37.0 ± 7.6	33.2 ± 7.1	0.1228
HR, bpm	63.8 ± 6.8	62.1 ± 7.2	63.9 ± 9.1	63.2 ± 8.7	0.8832
SBP, mmHg	129.1 ± 16.7	131.8 ± 16.8	129.6 ± 20.3	129.0 ± 18.3	0.9546
DBP, mmHg	76.0 ± 8.0	78.6 ± 12.3	76.8 ± 8.8	79.4 ± 11.9	0.7153
cfPWV, cm/s	1044 ± 103	1137 ± 190	1092 ± 226	1096 ± 163	0.5721
β‐stiffness, A.U.	15.4 ± 3.0	16.7 ± 3.1	15.2 ± 2.5	15.9 ± 1.5	0.3086
Total cholesterol, mg/dL	244.4 ± 49.1	252.2 ± 39.6	242.9 ± 36.3	251.4 ± 34.1	0.8398
HDL cholesterol, mg/dL	80.0 ± 23.8	82.4 ± 19.8	83.3 ± 19.2	86.5 ± 13.0	0.7753
Triglycerides, mg/dL	103.0 ± 40.2	93.0 ± 39.6	101.6 ± 36.3	91.3 ± 49.6	0.7458
Ang II, pg/ml	9.1 ± 8.3	9.3 ± 8.0	7.6 ± 3.9	8.7 ± 6.1	0.8638
ET‐1, pg/ml	1.6 ± 0.6	1.7 ± 0.4	1.6 ± 0.5	1.6 ± 0.5	0.9296
C1q, μg/ml	48.5 ± 11.2	41.8 ± 16.7	43.0 ± 11.0	48.7 ± 15.5	0.2511
Creatinine, mg/dL	0.8 ± 0.2	0.8 ± 0.1	0.8 ± 0.2	0.8 ± 0.2	0.7951
PRA, ng/ml	1.0 ± 0.8	0.8 ± 0.9	0.9 ± 1.0	0.9 ± 0.8	0.9282

Values are means and SD.

1‐RM, one repetition maximum; A.U., arbitrary units; Ang II, angiotensin II; AT, anterior thigh; C1q, complement component 1q; cfPWV, carotid‐femoral pulse wave velocity; CON, control; CSA, cross sectional area; DBP, diastolic blood pressure; EI, echo intensity; ET‐1, endothelin‐1; HDL, high‐density lipoprotein; HP, high‐protein intake; HR, heart rate; PRA, plasma renin activity; PT, posterior thigh; QF, quadriceps femoris; RF, rectus femoris; RT + HP, resistance training with high‐protein intake; RT, resistance training; SBP, systolic blood pressure; VL, vastus lateralis.

### Comparison of percent changes in body weight, muscle mass, muscle strength, and muscle quality before and after each intervention among the four groups

**Figure 2:**
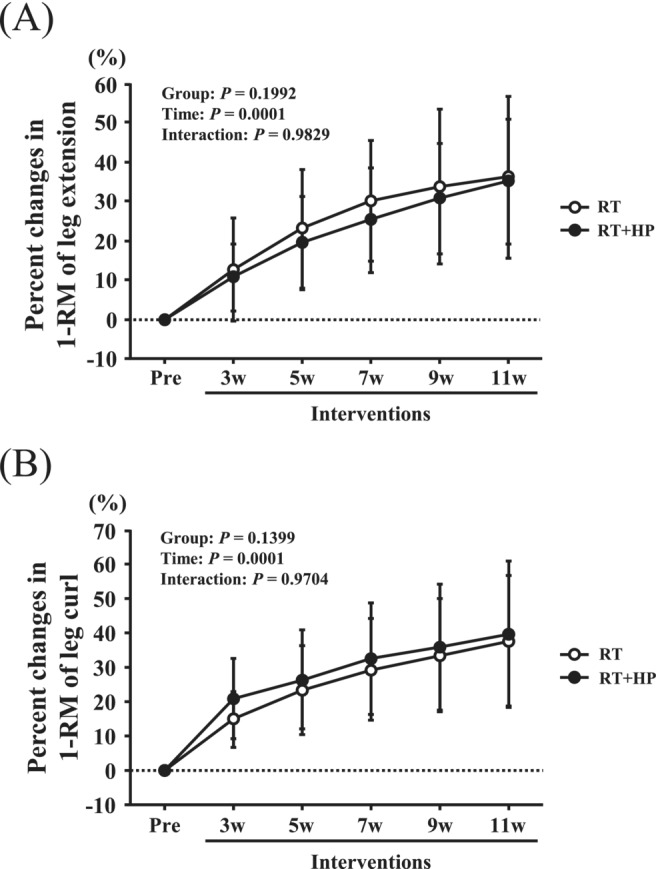
Time course of percent changes in one repetition maximum (1‐RM) of leg extension (A), and leg curl (B), measured at 2‐week intervals during a 12‐week resistance exercise intervention in the resistance training (RT) and combination of RT + HP (RT + HP) groups. Data are expressed as means ± SD.

There were no significant differences in percent changes in body weight among the four groups (Table 2). Percent changes in muscle thickness in the AT and muscle thickness in the PT in the RT + HP and HP groups were significantly greater than those in the CON and HP groups (*P* < 0.05, Table 2). The percent changes in muscle thickness in PT in the HP group were significantly greater than those in the CON group (*P* < 0.05; Table 2). Percent changes in the quadriceps muscle CSA in the RT + HP group were significantly greater than those in the CON, HP and RT groups (*P* < 0.05, Table 2), and percent changes in the quadriceps muscle CSA between the RT and CON groups were marginally significant (*P* = 0.09, Table 2). Percent changes in EI in the RF in the RT group were significantly lower than those in the CON and HP groups, and the percent changes in EI in the RF in the RT + HP group were significantly lower than those in the CON group (*P* < 0.05, Table 2), while percent changes between the RT + HP and HP groups were marginally significant (*P* = 0.05, Table 2). The percent changes in EI in the VL in the RT and RT + HP groups were significantly lower than those in the CON and HP groups (*P* < 0.05, Table 2). Percent changes in EI in the QF in the RT group were significantly lower than those in the CON and HP groups (*P* < 0.05, Table 2), and the percent changes in EI in the QF in the RT + HP group were significantly lower than those in the CON group (*P* < 0.05, Table 2). There were no significant interactions between group and time on percent changes in 1‐RM of leg extension and leg curl. However, significant main effects of time of percent change in 1‐RM of leg extension (Group: *P* = 0.1992, Time: *P* = 0.0001, Interaction: *P* = 0.9829, Figure 2A) and leg curl (Group: *P* = 0.1399, Time: *P* = 0.0001, Interaction: *P* = 0.9704, Figure 2B) were observed. The percent changes in the 1‐RM of leg extension and leg curl were significantly elevated after both RT and RT + HP (*P* < 0.05, Figure 2A,B).

**Table 2:** Comparison of percent changes in muscle mass and quality among the four groups

	CON	HP	RT	RT + HP	*P* value
Δ Body weight, %	−0.1 ± 2.7	−0.2 ± 2.3	−0.3 ± 2.4	0.6 ± 2.1	0.6511
Δ Muscle thickness in AT, %	−3.0 ± 8.2	−4.1 ± 8.2	13.7 ± 13.6*, ^#^	14.4 ± 15.1*, ^#^	0.0001
Δ Muscle thickness in PT, %	−2.0 ± 3.7	1.8 ± 4.8*	7.7 ± 6.0*, ^#^	7.7 ± 5.5*, ^#^	0.0001
Δ Quadriceps muscle CSA, %	1.3 ± 4.2	1.9 ± 2.6	3.6 ± 1.8^+^	6.6 ± 5.7*, ^#^, ^†^	0.0021
Δ EI in RF, %	10.1 ± 21.9	3.2 ± 17.9	−11.4 ± 12.8*, ^#^	−7.6 ± 11.5*, ^§^	0.0004
Δ EI in VL, %	9.0 ± 11.1	2.7 ± 13.8	−8.1 ± 11.2*, ^#^	−7.9 ± 17.1*, ^#^	0.0001
Δ EI in QF, %	8.7 ± 12.5	2.6 ± 12.9	−10.6 ± 8.8*, ^#^	−4.7 ± 20.2*	0.0002

Values are means and SD.

AT, anterior thigh; CON, control; CSA, cross sectional area; EI, echo intensity; HP, high‐protein intake; PT, posterior thigh; QF, quadriceps femoris; RF, rectus femoris; RT + HP, resistance training with high‐protein intake; RT, resistance training; VL, vastus lateralis.

* *P* < 0.05, vs. CON.

^#^ *P* < 0.05, vs. HP.

^†^ *P* < 0.05, vs. RT.

^+^ *P* = 0.09, vs. CON.

^§^ *P* = 0.05, vs. HP.

### Comparison of percent changes in cardiovascular disease risks before and after each intervention among the four groups

**Figure 3:**
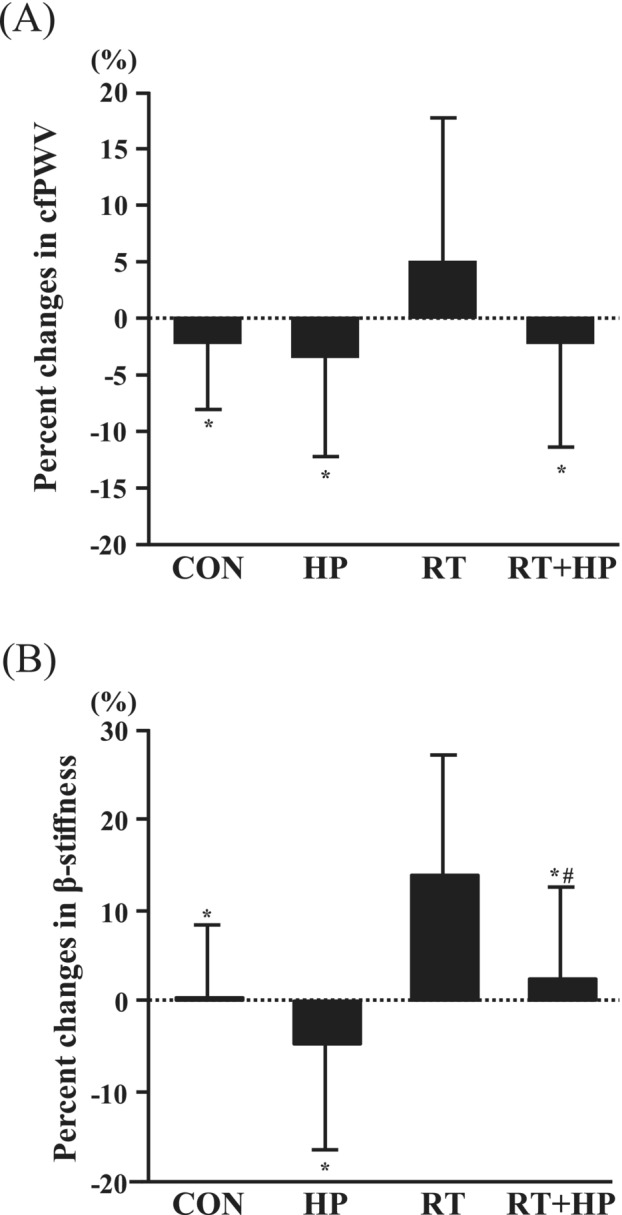
Comparison of percent changes in carotid‐femoral pulse wave velocity (cfPWV; A) and carotid β‐stiffness (B) before and after each intervention among the sedentary control (CON), high‐protein intake (HP), resistance training (RT), and combination of RT + HP (RT + HP) groups. Data are expressed as means ± SD. *P < 0.05 vs. RT. ^#^ *P* = 0.05 vs. HP.

There were no significant differences in the percent changes in HR, SBP, and DBP among the four groups (Table 3). The percent changes in cfPWV in the RT group were significantly greater than those in the CON, HP, and RT + HP groups (*P* < 0.05, Figure 3A). The percent changes in carotid β‐stiffness in the RT group were significantly greater than in the CON, HP and RT + HP groups, and furthermore, the percent changes in carotid β‐stiffness between the HP and RT + HP groups were marginally significant (*P* = 0.05, Figure 3B).

**Table 3:** Comparison of percent changes in cardiovascular disease risks among the four groups

	CON	HP	RT	RT + HP	*P* value
Δ HR, %	−9.0 ± 11.0	−6.1 ± 4.9	−7.6 ± 15.6	−5.5 ± 8.5	0.7373
Δ SBP, %	−1.4 ± 8.6	0.9 ± 12.0	−0.7 ± 10.0	−0.1 ± 13.1	0.9149
Δ DBP, %	−5.4 ± 7.6	−0.1 ± 8.8	−3.6 ± 9.3	−2.7 ± 8.6	0.2342

Values are means and SD.

CON, control; DBP, diastolic blood pressure; HP, high‐protein intake; HR, heart rate; RT + HP, resistance training with high‐protein intake; RT, resistance training; SBP, systolic blood pressure.

### Comparison of percent changes in blood biochemical markers before and after each intervention among the four groups

**Figure 4:**
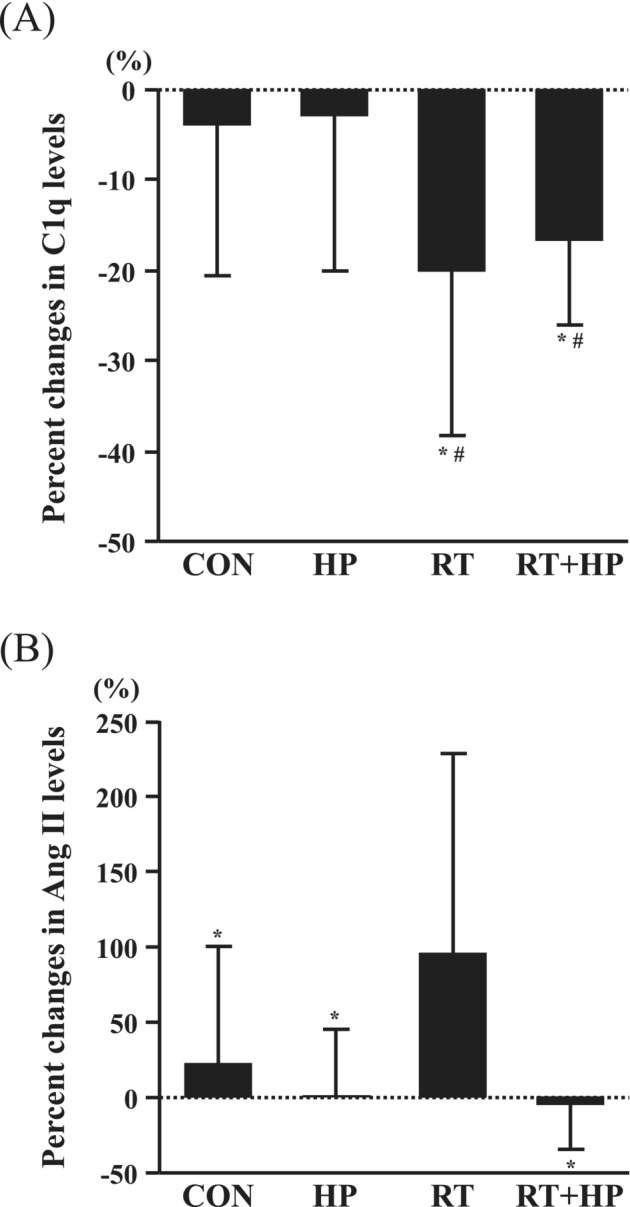
Comparison of percent changes in complement component 1q (C1q; A) and angiotensin II (Ang II; B) before and after each intervention among the sedentary control (CON), high‐protein intake (HP), resistance training (RT), and combination of RT + HP (RT + HP) groups. Data are expressed as means ± SD. *P < 0.05 vs. CON. ^#^ *P* < 0.05 vs. HP.

There were no significant differences in the percent changes in circulating levels of total cholesterol, HDL cholesterol, triglycerides, ET‐1, creatinine, and PRA among the four groups (Table 4). Percent changes in circulating C1q levels in the RT and RT + HP groups were significantly lower than those in the CON and HP groups (*P* < 0.05, Figure 4A). Percent changes in circulating Ang II levels in the RT group were significantly greater than those in the CON, HP, and RT + HP groups (*P* < 0.05, Figure 4B).

**Table 4:** Comparison of percent changes in blood biochemical parameters among the four groups

	CON	HP	RT	RT + HP	*P* value
Δ Total cholesterol, %	−5.2 ± 11.9	−7.7 ± 7.1	0.5 ± 14.8	−5.2 ± 9.0	0.1195
Δ HDL cholesterol, %	−4.0 ± 16.5	−4.6 ± 8.7	−5.5 ± 11.7	−2.5 ± 12.6	0.8988
Δ Triglycerides, %	5.5 ± 38.7	6.6 ± 28.3	15.0 ± 65.3	10.3 ± 30.4	0.8957
Δ ET‐1, %	7.8 ± 18.8	7.9 ± 27.2	−0.5 ± 13.0	1.9 ± 31.3	0.6041
Δ Creatinine, %	−1.5 ± 17.0	−2.7 ± 16.1	0.7 ± 15.5	6.0 ± 24.8	0.4760
Δ PRA, %	−6.0 ± 28.5	−4.6 ± 74.1	2.9 ± 76.3	−16.4 ± 53.5	0.8391

Values are means and SD.

CON, control; ET‐1, endothelin‐1; HDL, high‐density lipoprotein; HP, high‐protein intake; PRA, plasma renin activity; RT + HP, resistance training with high‐protein intake; RT, resistance training.

### Correlations of percent changes in circulating complement component 1q levels and muscle mass, muscle strength, and muscle quality

Significant negative relationship between the percent changes in circulating C1q levels and the percent changes in muscle thickness in AT (*r* = −0.427, *P* = 0.0001) were observed. Furthermore, there were significant positive relationships between the percent changes in circulating C1q levels and the percent changes in EI in RF (*r* = 0.270, *P* = 0.0153), the percent changes in EI in VL (*r* = 0.302, *P* = 0.0061), and the percent changes in EI in QF (*r* = 0.356, *P* = 0.0011).

### Correlations of percent changes in circulating angiotensin II and endothelin‐1 levels and arterial stiffness

Significant positive relationships between the percent changes in circulating Ang II levels, and the percent changes in cfPWV (*r* = 0.438, *P* = 0.0005) and carotid β‐stiffness (*r* = 0.328, *P* = 0.0068) were observed. There were no significant relationships between the percent changes in circulating ET‐1 levels, and the percent changes in cfPWV and carotid β‐stiffness.

## Discussion

This study investigated whether the combination of moderate to high‐intensity resistance training and regular intake of high‐protein food could improve arterial stiffness, muscle mass, strength, and quality in elderly women. In elderly women, resistance training increased the index of arterial stiffness despite improved muscle mass, strength, and quality, whereas a combination of resistance training and high‐protein intake did not significantly affect the index of arterial stiffness despite improved muscle mass, strength, and quality. Therefore, for the first time, a combination of moderate to high‐intensity resistance training and regular intake of steamed chicken breast increased muscle mass, strength, and quality without increasing arterial stiffness in elderly women.

It has been reported that a 10‐week lower limb strength training program increases circulating Ang II levels.^17^ As in a previous study, resistance training increased circulating Ang II levels. Additionally, the resistance training‐induced increase in circulating Ang II levels was positively correlated with changes in arterial stiffness. Thus, the resistance training‐induced increase in circulating Ang II levels may be associated with increased arterial stiffness. Meanwhile, we need to consider why the regular intake of high‐protein food inhibits the increase in arterial stiffness caused by resistance training. Ang II primes the premature senescence of vascular smooth muscle cells, but treatment with whey protein attenuates the Ang II‐induced effects.^18^ In vitro study has shown potent inhibition of angiotensin‐converting enzyme (ACE), which produces Ang II, in separate caseins and whey proteins of goat milk.^19^ Additionally, the whey peptide isoleucine‐tryptophan effectively inhibits ACE activity in the aorta and prevents age‐associated increases in aortic stiffness.^20^ Furthermore, 4‐week oral L‐histidine treatment decreased the aortic expression of ACE mRNA in spontaneously hypertensive rats.^21^ Ang II induces muscle atrophy, whereas supplementation with branched‐chain amino acids, which are abundantly present in chicken breast, ameliorates this effect.^22^ Thus, resistance training and/or regular intake of high‐protein food‐induced changes in circulating Ang II levels may be associated with vascular function in elderly women.

A previous meta‐analysis, focuses on moderate‐to‐high‐intensity resistance training, concluded that high‐intensity resistance training was associated with an increase in arterial stiffness; however, moderate‐intensity resistance training was not.^23^ Thus, resistance training intensity is a key variable for increasing arterial stiffness. Because the resistance training in this study was of moderate to high‐intensity, the changes in the indices of arterial stiffness in the RT group were greater than those in the CON group. Additionally, the effects of resistance training on arterial stiffness differ with age.^24^ These differences in the effects of resistance training on arterial stiffness from several research trials may be due to variances in resistance training protocols (frequency, intensity, sets, repetitions, and duration) and/or inclusion criteria and characteristics of participants (age, sex, body weight, and health status).^25^ Indeed, given the dose–response relationship between the increase in muscle strength and training intensity, moderate to high‐intensity resistance training elicits greater gains in muscle strength than low‐intensity resistance training.^26^ Moderate to high‐intensity resistance training as an exercise therapy is important for maintaining the health status of muscles and preventing sarcopenia in elderly individuals. Therefore, new strategies are required to address both age‐induced muscle loss and the resulting increase in arterial stiffness, such as those combined with chicken intake.

Steamed chicken breast was used as a high‐protein food in this study, and the regular intake of steamed chicken breast cancelled the resistance training‐induced increase in arterial stiffness. However, it is unclear which nutrients in steamed chicken breast contribute to this effect. Steamed chicken breast contains several amino acids and vitamins, such as histidine, glutamic acid, leucine, tyrosine, folate, vitamin K, vitamin E, vitamin A, and vitamin B. Higher intakes of glutamic acid, leucine, and tyrosine from animal sources were associated with lower PWV in female adults.^11^ Low vitamin K status is associated with increased vascular calcification,^27^ and vitamin K1 supplementation slows coronary artery calcification in healthy older adults.^28^ Additionally, the long‐term use of vitamin K2 supplements improves arterial stiffness in healthy postmenopausal women, especially those with high arterial stiffness.^29^ An association between high intake of vitamin E and a lower risk of CVD was observed in 87,245 middle‐aged women^30^ and 39,910 middle‐aged and older men.^31^ A meta‐analysis revealed that supplementation with vitamins A, C, and E reduced arterial stiffness.^32^ The regular intake of folate and methylcobalamin, the active form of vitamin B12, decreases arterial stiffness.^33^ Thus, these histidine, glutamic acid, leucine, tyrosine, folate, vitamin K, vitamin E, vitamin A, and vitamin B, all components of steamed chicken breast used in this study, may contribute to the decreased resistance training‐induced increase in arterial stiffness. Additionally, high‐protein intake did not significantly change PRA and serum creatinine levels, suggesting that high‐protein intake did not affect renal function in this study. Therefore, the high‐protein diet in this study was within the normal range of protein intake. However, the participants of this study were solely female older adults. Further studies are needed to examine the effects of a high protein diet and resistance training among male older adults.

Muscle mass, muscle strength, muscle quality, and circulating C1q levels of the participants in this study changed after both the RT and RT + HP interventions, whereas there were no significant changes in these several muscle indicators and circulating C1q levels between the two groups. Additionally, changes in circulating C1q levels were significantly correlated with changes in muscle thickness and EI. Our previous studies suggested that the decrease in circulating C1q levels by resistance training in the elderly is associated with resistance training‐induced increases in muscle mass and strength^34^ and that the decrease in circulating C1q levels by resistance training in aged mice contributes to the prevention of muscle fibrosis and atrophy via the attenuated muscle Wnt signaling pathway.^35^ Hence, in this study, the decrease in circulating C1q levels may have contributed to the improvement of muscle loss and quality after resistance training. However, the synergistic effects of high‐protein intake and resistance training were not observed in this study. In a previous study, 12‐week combined bodyweight resistance training and nutritional supplementation, containing protein and vitamin D, resulted in significantly greater improvements in muscle quality and strength in sarcopenic or dynapenic older adults compared to each group alone, suggesting a synergistic effect in older adults with skeletal muscle disorders.^36^ As the participants in this study were non‐sarcopenic and the exercise intensity was higher than that in the previous study, no significant synergistic effects on muscle mass, muscle strength, or muscle quality were observed in this study.

In conclusion, we showed that a 12‐week moderate to high‐intensity resistance training increased arterial stiffness and circulating Ang II levels in elderly women. Notably, regular intake of steamed chicken breast as a high‐protein food could cancel the resistance training‐induced increase in arterial stiffness. Furthermore, each intervention‐induced change in arterial stiffness was correlated with changes in circulating Ang II levels; thus, changes in the secretion of Ang II might be one of several mechanisms of resistance training with or without regular intake of high‐protein food‐induced changes in arterial stiffness. Needless to say, the muscle mass, strength, and quality were improved by both resistance training with/without regular intake of high‐protein food. Therefore, these results suggest that the combination of moderate to high‐intensity resistance training and regular intake of steamed chicken breast as a high‐protein food may be a novel exercise and nutritional therapy for elderly women.

## Conflict of interest

The authors declare no conflict of interest. M. Iemitsu received funding from the Ito Foundation.

## Funding

This study was funded by the Ito Foundation, Tokyo, Japan (#134, M. Iemitsu). This work was supported by Grants‐in‐Aid for Scientific Research from the Ministry of Education, Culture, Sports, Science, and Technology of Japan (KAKENHI: 22H03487 for M. Iemitsu).

## Supporting information


**Data S1.** Supporting Information
