# Supplementary material for: Impact of resistance training and chicken intake on vascular and muscle health in elderly women
Source: J Cachexia Sarcopenia Muscle. 2024 Nov 21;16(1):10.1002/jcsm.13572. doi: 10.1002/jcsm.13572 (PMC11670164; doi:10.1002/jcsm.13572)
Supplement: Supplementary file 1 — Data S1. Supporting Information [file JCSM-16--s001.pdf]

Supplemental Materials and Methods, *Data* S1

Materials and Methods

Measurement of body composition

The CSA of the quadriceps muscle was assessed using magnetic resonance imaging (MRI) (MAGNETOM Skyra, 3.0T, Siemens Healthineers, Erlangen, Germany), as previously described [S1]. For transverse MR acquisition of the quadriceps muscle, the participants lay supine and relaxed with synchronized respiration. We analyzed the anterior and lateral parts of the right thigh corresponding to the midpoint between the greater trochanter and the lateral condyle using image analysis software (sliceOmatic version 4.3 for Windows, Tomovision, Magog, QC, Canada). CSA was calculated as the ratio of the muscle area to the total femoral area.

Measurements of pulse wave velocity (PWV), blood pressures, and HR

The participants sat quietly for 30 min before the measurements were acquired. Resting brachial artery SBP, DBP, cfPWV (an index of arterial stiffness), and HR were measured in duplicate in the supine position using a vascular testing device (OMRON COLIN Co., Tokyo, Japan), as previously described [S2]. The cfPWV is the gold standard measurement of central arterial stiffness and is widely used in clinical practice. The cfPWV is considered as the representative PWV for the entire aorta. PWV was calculated from the time delay between the carotid and femoral artery blood pressure waveforms and the distance between the two points, which was measured using a non-elastic tape measure [S3]. The mean SBP and DBP values obtained from the left and right arms were calculated for the analysis.

Measurement of carotid β-stiffness

Carotid β-stiffness was examined as an indicator of arterial stiffness, as previously described [S4]. Before being tested, the participants sat quietly for 30 min. A combination of ultrasound imaging of the pulsatile common carotid artery and simultaneous applanation of tonometrically obtained arterial pressure from the contralateral carotid artery allowed noninvasive determination of carotid β-stiffness. Given that baseline blood pressure levels are subject to hold-down force, the pressure signal obtained by tonometry was calibrated by equating

the carotid mean arterial and diastolic blood pressures to the brachial artery value [S5]. The carotid β-stiffness index was calculated using the following equation: [ln (P1 / P0)] / [(D1 − D0) / D0], where D1 and D0 are the maximum (systolic) and minimum (diastolic) diameters, and P1 and P0 are the highest (systolic) and lowest (diastolic) blood pressures, respectively.

Measurement of muscle ultrasound

Muscle thickness and EI of the quadriceps were measured using ultrasonography, as described in previous studies [S6, S7]. Ultrasonography was performed after 15 min of rest to avoid the influence of body fluid shifts induced by muscle contraction. The participants were placed in a standing anatomical position, with their knee joints fully extended. We measured the anterior and posterior parts of the right thigh, corresponding to the midpoint between the greater trochanter and the lateral condyle. A real-time B-mode ultrasonography device (LOGIQ S7 Expert, GE HealthCare, Chicago, IL, USA) with a linear array probe was used to obtain images with the following acquisition parameters: anterior (frequency 8-10 MHz, gain 50 dB; depth, 8.0 cm) and posterior (frequency 8-10 MHz, gain 60 dB; depth, 10 cm) focus point 1 (top of the image). ImageJ software (Ver.1.48, National Institutes of Health, Bethesda, MD, USA) was used for the analysis. The thicknesses of the anterior and posterior thigh muscles were measured as the distances between the inferior edge of the superficial aponeurosis, which is located between the subcutaneous fat and skeletal muscle, and the superior edge of the femur. EI was assessed at the 256 gray scale level, which was expressed in arbitrary units (A.U.), using ImageJ software. Lower (darker) EI suggests better muscle quality [S8]. A rectangular region of interest that was as large as possible was established, excluding the visible fascia and bone in the RF and VL from the anterior image. The mean EI inside the region of interest in RF (EI in RF) and VL (EI in VL) was calculated for each image, and the mean EI from three images for each muscle was used for future analyses. We calculated EI in the QF using the following equation: EI in the QF = (EI in the RF + EI in the VL)/2.

Measurement of blood biochemical markers

Serum Ang II (RayBiotech Life, Inc., Peachtree Corners, GA, USA) and ET-1 (RayBiotech Life, Inc., Peachtree Corners, GA, USA) levels, as vasoconstrictor peptide hormones, and C1q (Hycult Biotech, Wayne, PA, USA), as an index of muscle fibrosis and

proliferation of vascular smooth muscle cells [S9, S10], and PRA (Abnova, Taipei, Taiwan) levels, as an index of renal function, were measured using an enzyme-linked immunosorbent assay (ELISA), according to the manufacturer's protocol. Serum creatinine levels, an index of renal function, were measured using a colorimetric assay (Cayman Chemical, Montgomery, TX, USA) according to the manufacturer's protocol. The optical densities of Ang II, C1q, and PRA in each sample at 450 nm and creatinine in each sample at 490 nm were measured using an xMark microplate reader (Bio-Rad Laboratories, Hercules, CA, USA). All the samples were converted into concentrations of the four parameters of each standard curve.

Measurements of serum cholesterol and triglyceride levels

Fasting serum total cholesterol, HDL cholesterol, and triglyceride levels were measured using standard enzymatic techniques. The measurements were performed by a subcontractor (medic, Shiga, Japan).

References

S1. Kishigami K, Kanehisa H, Qi S, Arimitsu T, Miyachi M, Iemitsu M, et al. Relationship between thigh muscle cross-sectional areas and single leg stand-up test in Japanese older women. *PLoS One.* 2022;17:e0269103.

S2. Fujie S, Sanada K, Hamaoka T, Iemitsu M. Time-dependent relationships between exercise training-induced changes in nitric oxide production and hormone regulation. *Exp Gerontol.* 2022;166:111888.

S3. Jeroncic A, Gunjaca G, Mrsic DB, Mudnic I, Brizic I, Polasek O, et al. Normative equations for central augmentation index: assessment of inter-population applicability and how it could be improved. *Sci Rep.* 2016;6:27016.

S4. Fujie S, Hasegawa N, Sato K, Fujita S, Sanada K, Hamaoka T, et al. Aerobic exercise training-induced changes in serum adropin level are associated with reduced arterial stiffness in middle-aged and older adults. *Am J Physiol Heart Circ Physiol.* 2015;309:H1642–H1647.

S5. Iemitsu M, Murakami H, Sanada K, Yamamoto K, Kawano H, Gando Y, et al. Lack of carotid stiffening associated with MTHFR 677TT genotype in cardiorespiratory fit adults. *Am Physiol Genomics.* 2010;42:259–65.

S6. Iemitsu K, Fujie S, Uchida M, Inoue K, Shinohara Y, Iemitsu M. Dioscorea esculenta Intake with resistance training improves muscle quantity and quality in healthy middle-aged and older adults: A randomized controlled trial. *Nutrients.* 2023;15:2438.

S7. Yoshiko A, Tomita A, Ando R, Ogawa M, Kondo S, Saito A, et al. Effects of 10-week walking and walking with home-based resistance training on muscle quality, muscle size, and physical functional tests in healthy older individuals. *Eur Rev Aging Phys Act.* 2018;15:13.

S8. Wong V, Spitz RW, Bell ZW, Viana RB, Chatakondi RN, Abe T, et al. Exercise induced changes in echo intensity within the muscle: a brief review. *Cell.* 2012;149:1298–313.

S9. Naito AT, Sumida T, Nomura S, Liu ML, Higo T, Nakagawa A, et al. Complement C1q activates canonical Wnt signaling and promotes aging-related phenotypes. *J Ultrasound.* 2020;23:457–72.

S10. Sumida T, Naito AT, Nomura S, Nakagawa A, Higo T, Hashimoto A. Complement C1q-induced activation of β-catenin signalling causes hypertensive arterial remodelling. *Nat Commun.* 2015;6:6241.
